# Desmosomal cadherins in zebrafish epiboly and gastrulation

**DOI:** 10.1186/1471-213X-12-1

**Published:** 2012-01-11

**Authors:** Alexander Goonesinghe, Xing-Ming Luan, Adam Hurlstone, David Garrod

**Affiliations:** 1 Faculty of Life Sciences, University of Manchester, (Michael Smith Building, Oxford Road), Manchester (M13 9PT), UK; 2 Rehabilitation Research Chair, King Saud University, P.O. Box 10219, Riyadh (11433)Kingdom of Saudia Arabia; 3 AstraZeneca, Zebrafish R&D, Brixham Environmental Lab, (Freshwater Quarry), Brixham, (TQ5 8BA), UK

## Abstract

**Background:**

The desmosomal cadherins (DCs), desmocollin (Dsc) and desmoglein (Dsg), are the adhesion molecules of desmosomes, intercellular adhesive junctions of epithelia and cardiac muscle. Both the DCs and desmosomes have demonstrably essential roles in mammalian development. In order to initiate their study in a more tractable developmental system we have characterised zebrafish DCs and examined their roles in early zebrafish development.

**Results:**

We find that zebrafish possess one Dsc, the orthologue of mammalian Dsc1, which we designate zfDsc. Unlike mammalian Dscs, zfDsc exists only as the "a" form since it lacks the alternatively-spliced mini-exon that shortens the cytoplasmic domain to produce the "b" form. Zebrafish possess two Dsgs, designated zfDsgα and zfDsgβ, orthologues of mammalian Dsg2. They show 43.8% amino acid identity and the α form has a 43 amino acid glycine-rich sequence of unknown function in its extracellular domain. Both zfDsc and zfDsgα were present as maternal and zygotic transcripts whereas zfDsgβ was first expressed from 8 hours post-fertilisation (hpf). All three transcripts were present throughout subsequent stages of development. Morpholino knockdown of both zfDsc and zfDsgα expression produced similar defects in epiboly, axis elongation and somite formation, associated with abnormal desmosomes or reduced desmosome numbers.

**Conclusions:**

These results demonstrate an important role for DCs and desmosomes in the early morphogenesis of the zebrafish embryo, provide a basis for more detailed analysis of their role and raise interesting questions relating to the evolution and functional significance of DC isoforms.

## Background

Cell-cell adhesion is a key mechanism that guides and co-ordinates the dynamic rearrangements of cell populations during animal development. Early zebrafish development is characterised by many such cell movements including epiboly, the spreading of the blastoderm over the yolk, ingression or involution at gastrulation, and convergence and extension, which lengthen the embryonic axis during and following gastrulation. Cell adhesion mediated by the Type 1 cadherin E-cadherin has been shown to play an essential co-ordinating role in each of these processes [1-5] and N-cadherin regulates posterior body formation [6-8].

Desmocollin (Dsc) and desmoglein (Dsg), the adhesion molecules of the intercellular junctions known as desmosomes, are also members of the cadherin superfamily [9-11]. Desmosomes maintain strong adhesion in adult epithelia and cardiac muscle but appear very early in mammalian development, for which their function is essential [12-15]. Ultrastructural evidence showed that desmosomes appear at the mid gastrula stage in the embryo of the teleost *Fundulus heteroclitus *and zebrafish [16,17] so desmosomes could also contribute to fish early development, but this has not been investigated.

The desmosomal cadherins are transmembrane proteins whose extracellular domains mediate principally homophilic adhesion in the desmosomal interspace or desmoglea [18]. Their cytoplasmic domains interact with linker or adaptor proteins plakoglobin, plakophilin and desmoplakin forming a dense desmosomal plaque, which is linked to the intermediate filament cytoskeleton, thus forming the desmosome-intermediate filament complex [19]. In mammals both Dsc and Dsg are present as multiple genetic isoforms that show differential tissue expression. For example in human tissues there are three Dscs and four Dsgs [20]. Dsc2 and Dsg2 are ubiquitous in tissues containing desmosomes but Dsc 1 and 3 and Dsg 1, 3, and 4 are largely confined to stratified epithelia where they show differentiation-specific expression [20,21]. Desmosomes in cells expressing multiple isoforms contain a mixture of those isoforms [18,22]. It is not clear why multiple isoforms of desmosomal cadherins are functionally necessary. Do they have specific adhesive functions or do they carry out specific roles in tissue differentiation and morphogenesis? The evidence from gene deletion and over-expression studies in mice suggests that they may have both adhesive and signalling functions (reviewed by [11,23]).

The three mammalian Dsc isoforms also show alternative splicing of their cytoplasmic domains giving rise to 'a' and 'b' forms. The mammalian genes contain seventeen exons of which exon 16 is small and contains a stop codon. When this is spliced in the cytoplasmic domain of the 'a' form, roughly equal in size to those of Type 1 cadherins, is truncated giving the shorted 'b' form. Although it is usually present in roughly equal quantity to the 'a' form, the function of the 'b' form is unknown. The 'a' form alone appears sufficient to support desmosomal plaque formation [24].

In order to initiate the study of the role of desmosomes in early zebrafish development we have cloned zebrafish Dsc and Dsg, studied the timing of their expression and carried out knock down of their expression. We find that the desmosomal cadherins have a role in zebrafish development from epiboly onwards.

## Methods

### Primer design

All primers for RT-PCR analysis of zfDsc, zfDsgα and zfDsgβ, and control primers were designed by Primer 3 program http://www.invitrogen.com/site/us/en/home/Products-and-Services/Product-Types/Primers-Oligos-Nucleotides/applied-biosystems-custom-primers-probes.html. The primers used for DSC1 were 5'-AAGGCGGTGTATGAGGTCAC-3' and 5'-GGTGCCTCTGTGTTGGATTT-3'; DSG2a 5'-CCAGTTCATGGTCATCGT-3' and 5'-GTCAGTGCAAAGTGTCTGG-3'; Dsg2b 5'-GGTGGAGGAAAACACCAGA-3' and 5'-GAGCATGGTGTCGCTGTCTA-3'; Beta-actin 5'-CCACGAGACCACCTTCAACT-3' and 5'-CATTGTGAGGAGGGCAAAGT-3'.

### Rapid amplification of cDNA ends (RACE)

Primers for both 5' and 3' RACE were also designed by Primer 3 program. The primers used for RACE were:- 5'RACE, **GSP 1**, CTG TCA AAA GCC TTG CCT TC; **GSP 2**, ATG GGT GAA AGG GGG CTT TG; **GSP 3**, ATC CAC AGC CCC AGG ATT CA; 3'RACE, **GSP**, GAC TGC GGG GAA ATG GAC AG; **GSP 1**, TAG GAG CTG CGG GTT TCC TG. zfDsgα 5' sequences were obtained using the 5' RACE kit according to the manufacturer's instructions. Three antisense primers (GSP 1-3) were made based on the previously determined zfDsgα sequence. GSP 1 (Gene specific primer) was used for the first strand cDNA synthesis and GSP 2 and 3 were used for nested PCR. The PCR conditions of GSP 2 and GSP 3 were the same, with the cycling numbers of 35 and annealing temperature of 58°C. The 3' end of the zfDsgα was amplified using the 3' RACE kit according to the manufacturer's instructions. Two sense primers (GSP and GSP 1) for nested PCR were designed based on the known 3' sequence. Cycling numbers of GSP was 35 with the annealing temperature of 58°C, and cycling numbers of GSP 1 was 35 with the annealing temperature of 60°C.

### Sequencing and bioinformatics

The ABI PRISM BigDye Terminator Cycle Sequencing Ready-reaction Kit was used for DNA sequencing as per the manufacturer's instructions. The sequencing reactions were processed in the Central Sequencing Facility (Faculty of Life Sciences, University of Manchester) using the AB377 sequencer (Applied Biosystems). The data from the sequencing were analyzed using BioEdit software. Zebrafish EST and YAC clone sequences were retrieved by searching the databases of NCBI http://www.ncbi.nlm.nih.gov/ and ZFIN http://zfin.org. ENSEMBL http://www.ensembl.org/ was used to predict gene structure and give a predicted cDNA, genomic and protein sequences. The chromosome location of zfDsc and zfDsgα was analyzed by ZFIN, VEGA http://vega.sanger.ac.uk/Danio_rerio/ and Tübingen http://wwwmap.tuebingen.mpg.de/. NCBI Blastp program and Workbench http://seqtool.sdsc.edu/CGI/BW.cgi/ were used to analyze protein homology, and NCBI Blastn program was used to analyze nucleotide homology. Ebi http://www.ebi.ac.uk and Expasy http://us.expasy.org were used to predict the peptide sequence of cDNA sequences. PSORT II http://psort.hgc.jp/form2.html was used to predict the signal peptide.

### PCR amplification and electrophoresis

1 μl template cDNA was added to a mixture of 2 μl MgCl_2 _(25 mM, Promega), 2 μl 10× Buffer (Promega), 0.5 μl dNTPs (Bioline), 0.5 μl each of forward and reverse primers and 0.2 μl Taq polymerase (Promega). The final reaction mixture was made up to a volume of 20 μl with ddH_2_O, the reaction mixture was amplified in a thermal cycler using standard cycling conditions. In all PCR reactions a minus template control was used as a genomic contamination control.

The DNA in solution (mixed with 5× loading dye) and either Hyperladder I or IV (Bioline) were both run on a TAE agarose gel (containing ethidium bromide at 10 mg/ml) at varying concentrations depending on product size (< 100 bp on a 2% gel to > 10 KB in a 0.8% gel) in 1× TAE buffer at 15 V/cm of gel. The different bands were visualized by UV illumination and photographed. Comparison of the product band to Hyperladder I/IV™ (Bioline) allowed the determination of the product size.

### RNA extraction, cDNA and 'rescue' mRNAsynthesis

Embryos in their experimental groups (50 embryos) were homogenised in 1 ml Trizol (Invitrogen) using a Teflon homogeniser. 200 μl chloroform was added to the samples and they were shaken vigorously and incubated for 2-3 minutes at RT, then centrifuged for 15 minutes at 12,000 × g at 4°C. The aqueous phase was removed into a fresh tube, 500 μl isopropanol was added and the samples were incubated for 10 minutes at RT and centrifuged for 10 minutes at 12,000 × g at 4°C. The supernatant was removed, the pellet was washed with 1 ml 75% ethanol and centrifuged for 5 minutes at 7,500 × g at 4°C. The ethanol was removed and the pellet was air dried and re-suspended in 40 μl RNase-free water, if the pellet had trouble dissolving the solution was incubated for 5 minutes at 60°C. The concentration of RNA was determined by using a NanoDrop™ spectrophotometer.

The Qiagen Omniscript cDNA synthesis kit and its components were used to synthesise cDNA from RNA samples. 1 μg RNA was added to 2 μl 10X RT Buffer, 2 μl dNTPs (0.5 μM each), 0.5 μl RND primers (Invitrogen), 0.5 μl of OligodTs (Invitrogen), 1 μl RNase inhibitor 10 units/μl, (Roche), 1 μl Omniscript RT and made up to 20 μl with RNase-free water. This was incubated at 37°C for 1 hour and then stored at -20°C.

The Ambion T7 mMessenger™ kit was used to generate 5' capped mRNA from the pT7Ts vector. These were diluted to the appropriate concentration before being co-injected with the corresponding morpholino.

### *In situ *hybridisation probe synthesis

To synthesize the probe a mixture of 2 μl linearised DNA (μg), 2 μl 10× transcription buffer, 2 μl DIG labelling mixture (Roche), 1 μl (20 units) of RNase inhibitor and 2 μl (40 units) T7 RNA polymerase made up to 20 μl with RNase free water was incubated for 2 hours at 37°C. Afterwards 1 μl of RNase free DNase I™ (Roche) was added and incubated for further 30 minutes and then the reaction was cleaned up using the Qiagen RNA easy mini kit as per manufacturer's instructions. The probe was then diluted 1:1 with formamide and stored at -80°C.

### *In situ *hybridisation

Fixed de-chorionated embryos were incubated for 4 hours in approximately 1 ml HYB buffer at 60°C. The HYB buffer was replaced with a mixture up to 500 μl of preheated HYB buffer and the corresponding probe (0.5 - 2.5 ng/μl). The embryos were then incubated o/n at 60°C. The next day the embryos were washed briefly in HYB wash buffer and then put through a series of 15 minute washes in mixtures of HYB buffer and SSC in different compositions (75% HYB buffer/25% 2 × SSC, 50% HYB buffer/50% 2 × SSC, 25% HYB buffer/75% 2 × SSC, 100% 2 × SSC) at 60°C, the embryos were then washed 2 × 30 minutes in 0.2 × SSC at 60°C before they were then put through a further series of 5 minute washes with a mixture of 0.2 × SSC and PBST (75% 0.2 × SSC/25% PBST, 50% 0.2 × SSC/50% PBST, 25% 0.2 × SSC/75% PBST). After this the embryos were washed 2 × 5 minutes in PBST. The embryos were then put in PBST with 2% blocking reagent for a minimum of 1 hour at RT, they were incubated for o/n at 4°C in blocking reagent with the diluted antibody, anti-dig (1.5 μl in 5000 μl blocking reagent).

The following day the embryos were washed 8 × 15 minutes in PBST and 3 × 5 minutes in freshly prepared NTMT solution, the embryos were then transferred into BM purple ready-to-use-solution (Roche) to perform the colour reaction.

Following the colour development under the microscope the colour reaction was stopped at the necessary point by washing the embryos 3 × 5 minutes with PBS. To reduce the background staining due to the BM purple the embryos were transferred through a series of glycerol/PBS-solutions (30 minutes in 20% glycerol/80% PBS, 30 min in 50% glycerol/50% PBS, then in 80% glycerol/20% PBS). Images were taken with a Zeiss Axioplan dissection microscope.

### Zebrafish husbandry and collection of embryos

Zebrafish were maintained according to standard conditions described in the zebrafish handbook, [25]. Embryos were cultured at 28.5°C in conditioned water (60 mg Instant Ocean salts, Tropic Marin^® ^in 1 litre deionised H_2_O).

TL *(Tübingen Longfin) *strain zebrafish embryos were used in all experiments. To generate embryos, adult females and males were transferred into breeding tanks (two females and two males per tank) the night before the embryos were required. Males and females were separated by a plastic divider. The following morning the dividers were removed and the fish allowed to breed. Embryos were collected 30 minutes after removal of the dividers and the fertilised ones used for injection.

### Micro-injection

Special injections plates were prepared for the injections -1.5% agarose in conditioned water was set in a 94 mm Petri-dish with an inserted (custom made) mould, which was removed after the agarose had set, to create special grooves to hold the embryos. The collected embryos were lined up in the grooves and injected (a volume of approximately 3 nl) with of RNA, MO or MO, by using a gas driven microinjection apparatus and glass needles. The injections were carried out into the yolk before the 16-cell stage of development. After that the embryos were incubated in fresh Petri-dishes with conditioned water at 28.5°C. All embryos were destroyed at 96 hpf in accordance with Home Office regulations.

### Morpholinos

Morpholinos were made up to a stock concentration of 5 mM in dd H_2_O. 2 nl were injected into each embryo at concentrations of 0.25 mM, 0.05 mM or 1 mM. Morpholino sequences are shown in Table 1.

**Table 1:** Morpholino sequences

Morpholino	DNA Sequence (5' - 3')
Dsc 1 ATG block	GCG TTC ATA CAT CCT GAA GCG AGA G

Dsc 1 '2' ATG block	GAT GTG TCC GGT CTC CAC CAT AAA C

Dsg 2a ATG block	AAC AGG TGA AAT TCG CCG GGC C

Dsg 2a '2' ATG block	CCG GTA GAA CAC GAT ATT TCC TGA T

Dsg 2a splice block	TGC AGG TCA CAT ACC AAC AGC ACT G

Control	CCT CTT ACC TCA GTT ACA ATT TAT A

### De-chorionation, observations and DIC analysis of live embryos

Embryos were incubated in a pre-warmed pronase (Fluka) solution (2 mg/ml) at 37°C for 20 minutes. Afterwards the chorions were removed by passing the embryos several times gently through a glass Pasteur pipette. They were then transferred to an 8× chorion wash solution three times.

Observations of live embryos were carried out in the chorions or alternatively the embryos were manually de-chorionated and mounted in a 1.5% solution of methyl cellulose in 8× chorion water, held in a specially designed embryo mould and viewed using either a Zeiss Stereolumar dissecting stereoscope or Zeiss AxioPlan with Nomarski optics. For description of the stages and timing of embryonic development see [26].

To categorise the MO injected embryos into phenotypes, the embryos were left overnight at 28.5°C. The categorisation was carried out into wildtype, mild, moderate and severe phenotypes. To take images of the observed phenotypes by using a compound microscope with Nomarski differential interference contrast (DIC) optics as well as a stereo-microscope at 24 hpf, the embryos were de-chorionated by incubating them for 15 min in pre-warmed pronase solution (2 mg/ml) at 37°C. Afterwards the embryos were anaesthetised using MS222 and held in 8 × chorion solution.

### SYTOX staining

SYTOX nuclear green stain is impermeable to living cells, but stains nuclei in a syncitium (or otherwise following membrane degradation). A 5 mM solution in DMSO was co-injected in a 50:50 mix with 1 mM MO.

### Electron microscopy

Embryos were fixed overnight in 2% paraformaldehyde/2% glutaraldehyde in 0.1 M cacodylate buffer at pH 7.4, then post-fixed in 1% OsO_4 _in the same buffer for 2 hours, dehydrated and embedded in Spurr resin. Ultrathin sections were stained with 1% uranyl acetate and 0.3% lead citrate, then examined on a FEI Technai Biotwin electron microscope.

## Results

### Isolation of zebrafish desmocollin and desmoglein sequences

The partial cDNA sequence for zebrafish Dsc (zfDsc) was obtained from four overlapping EST sequences from the TIGR Zebrafish Gene Index (Accession numbers: TC225818, TC130266, TC169171 and TC182641) and one EST sequence from GenBank (Accession number: CD283558.1) and used to obtain a predicted cDNA sequence from Ensembl. From this assembly, overlapping primer sets were designed to clone by PCR and sequence the full length zfDsc mRNA from 4 dpf zebrafish embryos. This yielded a cDNA of 3.4 kb with an ORF of 2676 bp encoding 892 amino acids (GenBank Accession number: JQ013460) (Additional file 1, Figure S1). All available EST sequences with homology to mammalian Dsc appeared to arise from a single gene.

A partial sequence for zfDsg was obtained from three overlapping TIGR EST sequences (Accession numbers: AI883817, AI397144 and BI878525) and used to predict the cDNA sequence from Ensembl. Five overlapping primer pairs were then used to obtain the sequence. The sequence obtained still lacked correct 5' and 3' ends so both ends were extended by RACE. Finally a full length cDNA of 3.6 kb was obtained containing an ORF of 3429 bp encoding a protein of 1143 amino acids (GenBank Accession number: JQ013461) (Additional file 1, Figure S2). This sequence was designated zfDsgα to distinguish it from a second sequence, designated zfDsgβ, indentified during nucleotide homology analysis from a NCBI EST sequence (Accession number: BI884774). A partial cDNA sequence for zfDsgβ was obtained by PCR (GenBank Accession number: JQ013462) (Additional file 1, Figure S3). These results indicate that zebrafish have one desmocollin isoform and two related desmoglein isoforms.

### Genomic organisation of zebrafish desmosomal cadherins

Searching the zebrafish genome in Ensembl (Zv9) revealed that zfDsc cDNA was located on chromosome 20, at position 7,437,162-7,468,565 (reverse strand) (ENSDARG00000039677), so the zfDsc gene is about 30 kb in size. Again this analysis suggested the existence of only a single Dsc paralogue in zebrafish. The gene for zfDsgα (ENSDARG00000062750) is also located on chromosome 20 at position 16,990,224-17,010,459 also on the reverse strand. The gene for zfDsgβ (ENSDARG00000076426) is located on chromosome 2 at position 2,392,229-2,424,395. No other Dsg paralogues were detected.

Analysis of intron-exon boundaries showed that the zfDsc gene comprises 16 exons compared with 17 for mammalian Dscs (Table 2). There is a high level of exon size conservation as exons 5, 8, 9 and 11 are the same size in zfDsc and all three human Dscs. The "missing" exon in zfDsc is the alternatively-spliced mini exon, exon 16 in the mammalian genes, which contains a stop codon and generates the cytoplasmically-truncated 'b' form of mammalian Dscs [27]. Exon 16 in zfDsc resembles exon 17 in mammalian Dscs, encoding the COOH-terminus of the longer 'a' form. That zfDsc has only the 'a' form was supported by the result of RT-PCR. Only a single band was amplified using the 3' primer set comparable to primers that produce two bands in mammals [27] (not shown). Thus, zfDsc lacks the 'b' form found in mammals.

**Table 2:** Comparison of zfDsc exon sizes with three human desmocollin genes

Exon	hDsc1	hDsc2	hDsc3	zfDsc	Protein domain
1	Non-coding +63	+69	+69	Non-coding +42	S

2	85	85	85	91	P

3	203	203	203	191	EC1
	
4	120	120	120	114	

5	***156***	***156***	***156***	***156***	EC2
	
6	145	145	145	142	

7	167	167	167	155	EC3
	
8	***135***	***135***	***135***	***135***	

9	***186***	***186***	***186***	***186***	EC4
	
10	260	257	257	248	

11	***143***	***143***	***143***	***143***	EA
	
12	213	225	225	222	

13	240	***237***	225	***237***	TM

14	122	125	122	116	CYT
	
15	249	258	258	285	

16	46	46	43	Non-coding +213	Alternative splicing

17	Non-coding +195	195	195	Non-coding +213	TD

The numbers in bold italics indicate exons having identical sizes. The right hand column shows the approximate corresponding protein domains. S = signal, P = precursor, EC = extracellular, EA = extracellular anchor, TM = transmembrane, CYT = cytoplasmic, TD = terminal domain of human "b" form.

The zfDsgα gene comprises 14 exons, one less than human and mouse Dsg 2 (Table 3). Exon sizes are not highly conserved although the sizes of exons 3, 7 and 9 in zfDsgα are equal to exons 4, 8 and 10 in human and mouse Dsg.

**Table 3:** Comparison of exon sizes of zfDsgα with those of human and mouse Dsg2

Exon	hDsg2	mDsg2	zfDsga	Protein domain
1	+45	+60	+54	S+P

2	36	36	129	EC1

3	135	174	***162***	
	
4	***162***	***162***	142	EC2
	
5	145	145	164	

6	167	167	132	EC3
	
7	138	138	***186***	

8	***186***	***186***	395	EA4

9	266	266	***143***	EA

10	***143***	***143***	222	TM

11	228	228	240	IA

12	228	228	119	ICS

13	122	122	297	LD

14	333	330	1044	RUD

15	+1020	+1020		TD

The numbers bold italics indicate exons having identical sizes. The right hand column shows the approximate correspondence with the protein domains. Abbreviations as for Table 1 and in addition ICS = intracellular cadherin-like segment, LD = linker domain, RUD = repeat unit domain.

### Zebrafish desmosomal cadherins are the orthologues of mammalian Dsc1 and Dsg2

**Figure 1:**
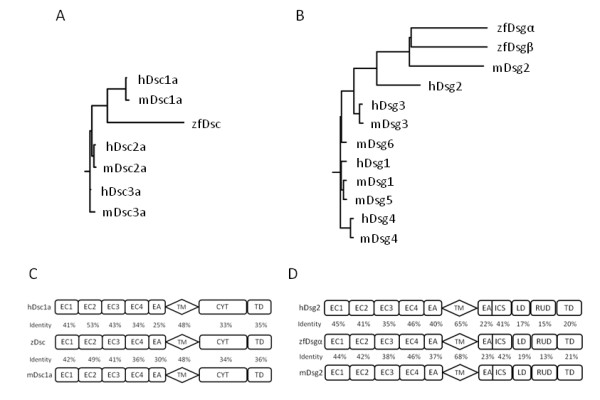
**Relationships between zebrafish and mammalian desmosomal cadherins**. A and B. Rooted homology trees outlining the predicted relationship between, and relative evolutionary distance of, zebrafish, human and mouse desmocollins (A) and desmogleins (B). C and D. Predicted domain homology of zfDsc (C) and zfDsga (D) against both relative human and mouse amino acid sequences. EC - extracellular domain, EA - extracellular anchor, TM transmembrane domain, CYT - cytoplasmic domain, TD - terminal domain, IA - intracellular anchor, ICS - intracellular catenin binding sequence, LD -, RUD - repeat unit domain. The sequences were form NCBI as follows: mouse Dsc1a (Accession no. CAA66628), Dsc2a (Accession no. AAA79177), Dsc3a (Accession no. NP_031908), human Dsc1a (Accession no. NP_077739), Dsc2a (Accession no. CAA40141), Dsc3a (Accession no. CAA58781); mouse Dsg1 (Accession no. Q61495), Dsg2 (Accession no. AAH34056), Dsg3 (Accession no. AAB65091), Dsg4 (Accession no. NP_853543), Dsg5 (Accession no. AAP31152), Dsg6 (Accession no. AAP31153), human Dsg1 (Accession no. CAA39976), Dsg2 Accession no. CAA81226), Dsg3 (Accession no. P32926), Dsg4 (Accession no. Q6W3BO).

Comparison of deduced amino acid sequences showed that zfDsc showed the highest homology with the human and mouse Dsc 1, with which it shared 68% and 70% amino acid homology, respectively (Figure 1A). Like the human and mouse proteins, zfDsc protein precursor comprises a 16 aa signal sequence followed by a 105 aa pre-protein. The mature protein has 771 aa and comprises four extracellular sub-domains (EC1 to EC4) followed by an EA domain and a 26 aa TM domain and a cytoplasmic domain equivalent to the mammalian Dsc1 'a' form. The cell adhesion recognition (CAR) site in EC1 of zfDsc is RAF instead of YAT in human and mouse Dsc1. Amino acid identity between the zebrafish and mammalian proteins is highest towards the NH_2_-terminus and, except for the TM domain, diminishes towards the COOH-terminus (Figure 1C).

Comparison of deduced amino acid sequences showed that zfDsgα to have the greatest homology with human and mouse Dsg 2, which shared 61% and 64% amino acid homology, respectively (Figure 1B). The zfDsgα protein precursor comprised a 16 aa signal sequence followed by a 21 aa pre-protein. Within the extracellular region, the high homology domains were EC1, 2 and 4. The CAR site in the EC1 domain is IAL rather than YAL in Dsg 2 of human and mouse. A glycine-rich protein sequence was found in the EC 3 domain. This showed no significant identity with any mammalian Dsg 2 or with any other protein in the databases. Its cDNA sequence did, however, show 100% identity to three zebrafish EST sequence (Accession number: BI881590.1, BE017197.1 and CK738901.1). The highest amino acid identity appeared in the zfDsgα TM domain, which shared 65% and 68% protein identity with human and mouse Dsg 2, respectively. The cytoplasmic region of zfDsgα was divided into IA, ICS, LD, RUD and TD sub-domain and, except the ICS region, the overall amino acid identity for this region was low (Figure 1D).

We were able to clone only part of the zfDsgβ sequence but were subsequently able to obtain a full length cDNA sequence from Ensembl (Accession number: ENSDART00000109073). The predicted amino acid sequence shows 43.8% identity with zfDsgα. We have used this sequence to generate the cladogram in Figure 1B. This suggests that zfDsgα and β are paralogues. Domain comparison between their predicted protein sequences is shown in Additional file 1, Figure S4.

### Desmosomal cadherins are continuously expressed from early in zebrafish development

**Figure 2:**
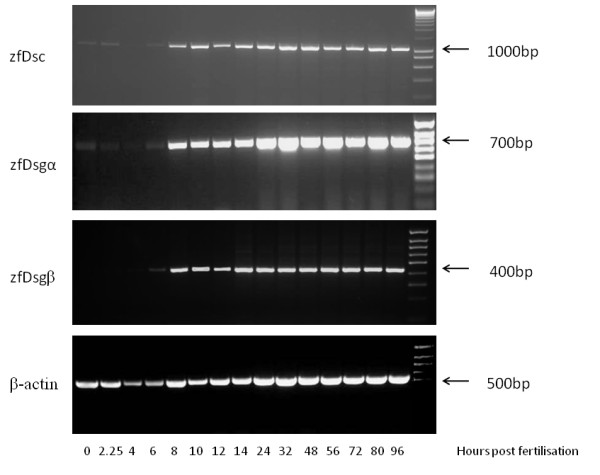
**Desmosomal cadherins are expressed from early development onwards**. Semi-quantitative RT-PCR analysis of zfDsc, zfDsga, zfDsgb and zfβ-actin expression through the first 96 hours of zf development. For further detail see text.

To determine the expression of each gene RT-PCR of mRNA from different stage embryos was performed using zfDsc primer pair 3, zfDsgα primer pair 4 and zfDsgβ primer pair 2. This showed that zfDsc and zfDsgα had the same expression pattern (Figure 2). Their transcripts were first identified at low levels in newly fertilized eggs (0 h) and in cleavage stages up to 128-cell stage (2^1^/_4 _hpf), declined to very low levels at the mid-gastrula period (4 hpf), but increased in the early gastrula period (6 hpf). Then they showed stronger expression from mid-gastrula period (8 hpf) to 96 hpf. These three phases of transcription probably indicates that both zfDsc and zfDsgα were present at first as maternal transcripts and then as embryonic transcripts. No maternal contribution was detected for zfDsgβ (Figure 2), which was first weakly expressed in the early gastrula period (6 hpf) and then increased its expression from the mid-gastrula period (8 hpf).

**Figure 3:**
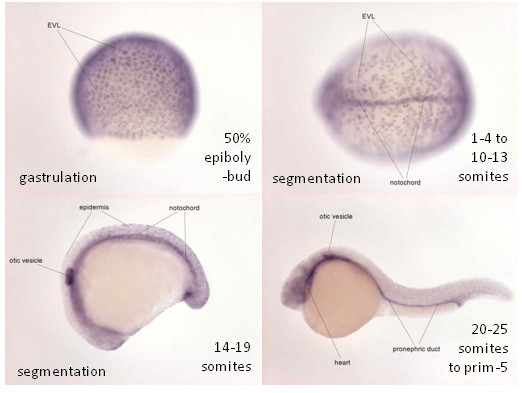
**Expression of zfDsc orthologue during zebrafish embryogenesis**. Expression of zfDsc revealed by WISH (purple colour). Note the prominent staining in the EVL during gastrulation, and subsequently in the notochord, otic vesicle, pronephric duct, heart and epidermis. Images courtesy of zfin http://zfin.org/action/marker/view/ZDB-GENE-031116-55.

Expression of zfDsc during zebrafish embryogenesis revealed by WISH was obtained from ZFIN (Figure 3) [28]. Note the prominent staining in the EVL during gastrulation, and subsequently in the notochord, otic vesicle, pronephric duct, heart and epidermis. Similar data for zfDsgs are currently unavailable.

### Knockdown of desmosomal cadherins affects epiboly, gastrulation and convergence-extension movements

**Figure 4:**
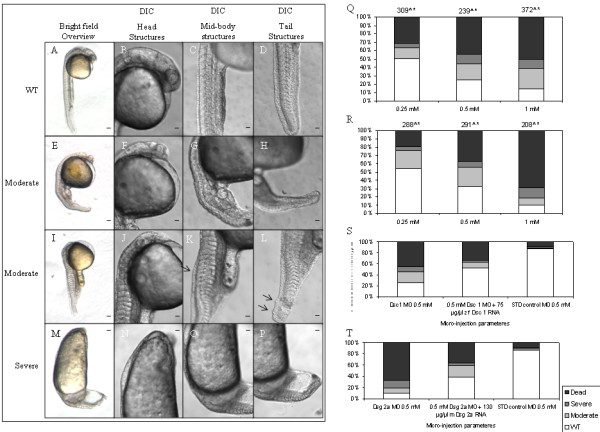
**Knock-down of individual desmosomal cadherins causes developmental defects**. The effects of morpholino knockdown of zfDsc and zfDsga as recorded at 24 hpf. As phenotypes for both morpholino knock-downs were very similar, representative images from their relative phenotypic classifications are shown in E-P. For comparison, WT animals are shown in A-D. Quantification of phenotypic distribution from morpholino knockdown of zfDsc and zfDsga are shown in Q and R, respectively. (N.B. Embryos that showed epiboly defects (see text and Figure 5) generally did not progress beyond 10 hpf and are therefore recorded as dead in this figure.) Quantification of standard control morpholino induced phenotypes and zfDsc/zfDsga knock-down embryos that have been partially rescued with corresponding Dsc1/Dsg2a mRNA are shown in S and T, respectively. Scale bars in A, E, I and M = 40 μm; other scale bars = 20 μm.

To elucidate the functions of zfDsc and zfDsg in zebrafish development MOs directed against the -75/+25 ATG region of the mRNA sequence (2 MOs per gene) and against the 3' splice donor sites of exon 2 in zfDsc and exon 4 in zfDsgα were injected into embryos between the 1 cell and 8 cell stages. (N.B. The Dsg MOs are complimentary to zfDsgα only.) In all cases the injected MOs caused defects in a dose-dependent manner. We could not distinguish between the phenotypes produced by MOs for zfDsc and zfDsgα. Defects included some embryonic lethality, usually due to abnormal epiboly, and phenotypic alteration of embryos that survived until 24 hpf. The phenotypes produced at 24 hpf were classified as wild type (WT), moderate, severe or dead (Figure 4). Features of the moderate phenotype included slight axis shortening, retarded head development, shortened and slightly bent tails, kinking of the notochord and altered somite morphogenesis (Figure 4E, F, G, H). The severe phenotype involved shortened body axis, severely reduced or absent head and or tail, absence of clearly defined somites and sometimes blebbing of the epidermis (Figure 4I, J, K, L, M, N, O, P). Because the effects of all ATG-directed MOs were similar the quantitative data are shown for the #1ATG MOs only (Figure 4Q, R). In order to show whether the phenotypes produced by MO injection could be rescued, mRNAs encoding full length zfDsc (75 ng/ml) and zfDsg α (130 ng/ml) - lacking however the MO target sequence by virtue of subcloning into pT7Ts - were injected into embryos that received the respective MOs at a concentration of 0.5 mM. This showed that the severity of the phenotypes produced was considerably reduced in each case (4 S, T).

**Figure 5:**
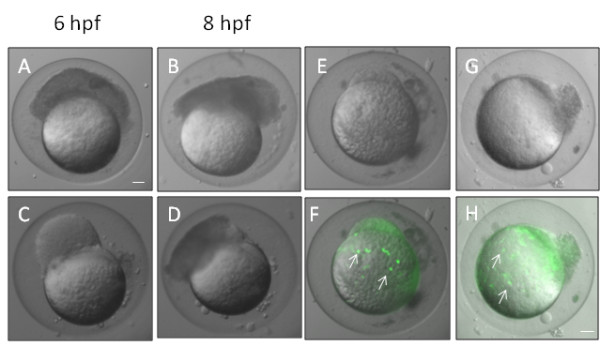
**Knockdown of desmosomal cadherins causes epiboly defects**. A and C show morphants at 6 hpf whose blastomeres became detached and failed to migrate towards the vegetal pole. The blastomeres of these embryos still continued to divide and seemed to undergo movements resembling gastrulation movements (B, D). These images are representative of similar phenotypes obtain by knockdown of both zfDsc1 and zfDsg2. E, F and G, H show embryos that were injected with Sytox to stain the nuclei of the YSL (white arrows in F, H). The DIC images (E, G) show that the blastomeres had become detached while the merged DIC and fluorescence images (F, H) show that the YSL had continued epiboly movement toward the vegetal pole. (Embryos such as those shown in this figure did not survive beyond about 10 hpf and are recorded as dead in Figure 3.) E, F were obtained by knockdown of Dsc1 and G, H by knockdown of Dsg2. Scale bars = 100 μm.

No abnormalities of cleavage were noted following MO injection, but the development of animals which would subsequently be dead by 24 hpf had arrested before 10 hpf with two clearly different phenotypes. Some embryos had a severe epiboly arrest with the DCs and EVL becoming detached from the external YSL (eYSL) that continued to undergo epiboly (Figure 5B, D). This left a population of cells at the animal pole which seemed to undergo cell division and attempt some gastrulation movements. Staining of eYSL nuclei with SYTOX appeared to show that the eYSL continued to show epiboly movements (5E, F, G, H). In the second arrest phenotype, the EVL was attached to the yolk (Figure 5A, C) but did not go through epiboly. It remained attached in a ring to the base of the blastoderm until, after extensive proliferation and blastoderm growth, the embryos arrested due to what is presumed to be mechanical strain of the EVL and detachment of the blastoderm from the yolk cell (Figure 5). Furthermore, those embryos that escaped epiboly arrest showed delayed epiboly. At 10 hpf, morphant embryos showed approximately 60% epiboly whereas STD control injected embryos had already completed epiboly.

**Figure 6:**
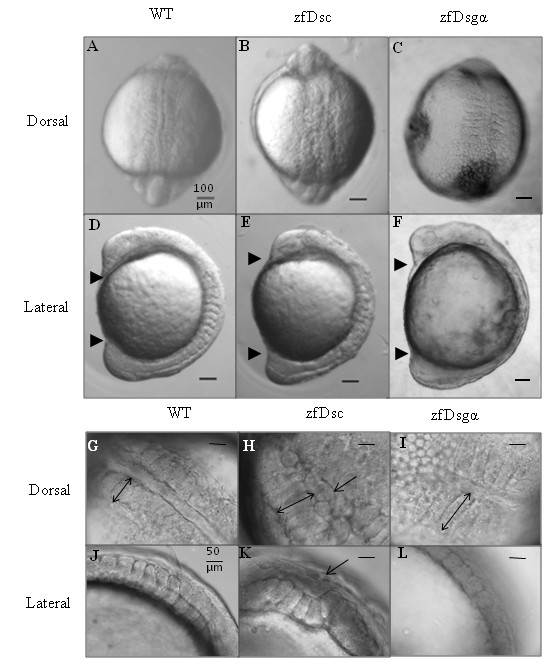
**Knockdown of desmosomal cadherins affects convergent-extension movements**. Representative DIC images of wild type and morphant embryos at 9-11 somites to illustrate convergent-extension defects produced by knocking down zfDsc1 and zfDsg2. A-C Dorsal views showing abnormal somite structure. D-F Lateral views showing shortening of embryonic axis (head and tail indicated by upper and lower arrowhweads, respectively). G-L Higher magnification pictures showing medio-lateral widening of somites (double headed arrows in G-I), kinking of notochord (arrows in H, K) and loss of chevron structure of somites (J-L). Scale bars = 100 μm (A-F) and 50 μm (G-L).

When embryos injected with either zfDsc or zfDsgα MOs reach later stages they exhibited morphological defects characteristic of those produced by altered gastrulation movements. These included a shorter embryonic axis, and undulating notochord and somites that were either altered in morphology or substantially disorganised (Figure 6). In some embryos the somites were medio-laterally extended compared with the wild type (Figure 6). These defects are consistent with altered convergent-extension movements during gastrulation.

**Figure 7:**
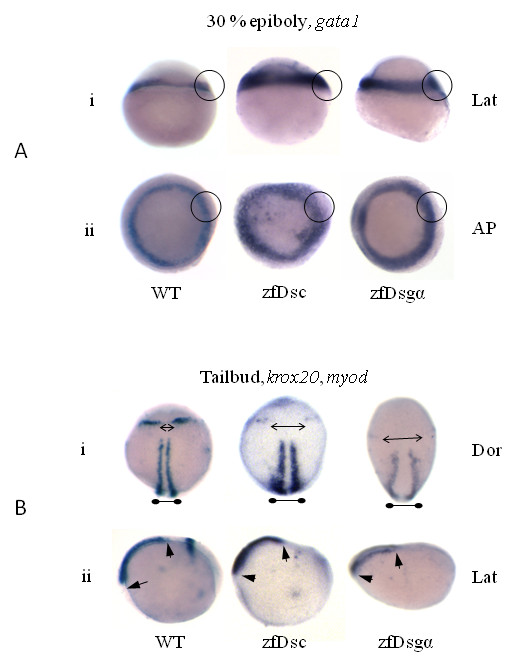
**Knockdown of desmosomal cadherins affects gastrulation movements**. *In situ *hybridisation images for *gata1 *at 30% epiboly (A) and *myod, krox20 *(B) at tailbud stage for both zfDsc1 and zfDsg2a morphant embryos. WT embryos are shown for comparison. A.i. and A.ii. show the lateral (Lat) and animal pole (AP) views, respectively. Circles indicate the expanded expression of gata1 in the germ ring margin of the morphants. B.i. and B.ii. show the dorsal (Dor) and lateral (Lat) views, respectively. Double headed arrows in B.i. indicate medio-lateral expansion of the distance between *krox20 *markers (pointed arrows) and *myod *marker (blunted arrows). Short arrows in B.ii show reduced rostro-caudal extension as indicated by the *myod *marker.

Altered gastrulation movements were also suggested by *in situ *hybridization studies with specific markers. Thus at 30% epiboly *gata1*, a mesodermal marker, showed larger areas of more diffuse expression in morphant embryos than in the wild type (Figure 7A). At the tailbud stage, both *krox20*, an ectodermal and rhombomere 3 and 5 marker, and *myoD*, a mesodermal and muscle marker, showed lateral divergence of the paraxial lines of staining (Figure 7B).

### Desmosome formation is reduced in morphant embryos

**Figure 8:**
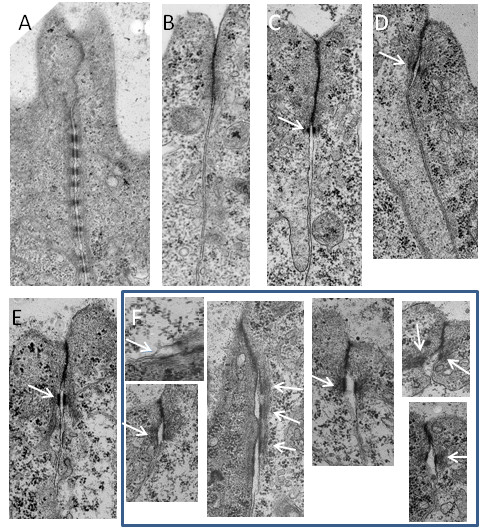
**Knockdown of desmosomal cadherins affects desmosome structure**. A. Apical junctional complex between cells of the EL of wild type embryo at 24 hpf showing the tight junction/adherens junction region (tj, aj) and the extensive array of desmosomes (d). At epiboly (B) the tight junction/adherens junction was present but no desmosomes were found. By the shield stages (C) fully formed desmosomes (arrow) were present forming the lowest component of the junctional complex in wild type embryos. D. A single fully formed desmosome (arrow) found in a morpholino injected embryo that reached the shield stage. At the 8 somite stage (E) desmosomes (arrow) were present at the lower aspect of the junctional complex in wild type embryos. F. A representative sample of abnormal desmosomes (arrows) form morpholino injected embryos that reached the 8 somite stage. Scale bars: A = 1 μm; B-F = 0.5 μm.

In the absence of reliable specific antibodies to use as desmosomal markers, wild type and embryos injected with the zfDsc MO [#1] were examined for desmosomes by electron microscopy. At 24 hpf the junctions between epidermal cells were abundantly supplied with desmosomes (Figure 8A). At epiboly no mature desmosomes were present. Instead cells of the enveloping layer possessed apical junctional complexes of adherens/tight junction type (Figure 8B). Similar junctions were also present in MO-injected embryos (not shown). At the shield stage fully formed desmosomes were present as the basal components of junctional complexes between EL cells (Figure 8C, arrow). However, in MO-injected embryos hardly any desmosomes were present. As there were so few this was difficult to quantify accurately but in scanning the entire peripheries of median sections of five embryos only one fully formed desmosome was encountered (Figure 8D, arrow) together with four possible desmosomes that were very poorly formed. Control embryos at the 8 somite stage possessed abundant fully formed desmosomes at the bases of junctional complexes between epidermal cells of embryos (Figure 8E, arrow). Desmosomes were also present in the same location in MO-injected embryos. However, many of these were poor in structure showing a wide intercellular space or unpaired plaques (Figure 8F, arrows). A count showed that approximately 70% of desmosomes in injected embryos were poorly formed whereas none of this type were present in controls. These results suggest that desmosome formation was both delayed and substantially abnormal in MO-injected embryos.

## Discussion

Our results show that the zebrafish possesses 3 desmosomal cadherin genes and that the desmosomal cadherins are of functional importance in embryonic development from epiboly and through gastrulation and axis formation.

The single zebrafish desmocollin gene shows two surprises. Firstly, it is the orthologue of mammalian Dsc1, which is principally expressed in the differentiated layers of stratified squamous epithelia, rather than the more widely expressed Dsc2. In mammals Dsc1 is involved in strengthening cell-cell adhesion in these epithelia, particularly epidermis [29], and, since it is the only desmocollin present, zfDsc presumably has a similar function wherever it is expressed. (Previous work has suggested the presence of Dsc2 in zebrafish and suggested that it has a role in heart development [30]. However, while this work appears to relate to the gene we describe here as zfDsc, it difficult to compare that work with ours due to the lack of information regarding methods and sequences in [24].)

Secondly, zfDsc lacks a shortened 'b' form, which in mammals is produced by alternative splicing of a mini exon [27], also missing from zebrafish. Thus zfDsc corresponds to the longer 'a' form of mammals. This is interesting because the functional significance of the 'b' form is not clear, the 'a' form having been shown to be sufficient for desmosomal plaque formation, while the 'b' form does not support plaque formation [24]. Apparently the zebrafish can form perfectly normal desmosomes in the completed absence of a 'b' form leaving the significance of the 'b' form even more in doubt.

There are two zebrafish desmoglein genes, both orthologues of the widely expressed mammalian Dsg2. Designated zfDsgα and zfDsgβ, they differ in that α contains a 40 amino acid, glycine-rich insert in its extracellular domain and share 43.8% amino acid identity. The insert is unique to zebrafish and of unknown functional significance. Both α and β are expressed early in zebrafish development though β is not represented in maternal transcripts. It may be suggested that both are of functional significance in early development, though this remains to be demonstrated for β.

It was a surprise to find that knock-down of desmosomal cadherins disrupted development from early epiboly because ultrastructural studies of developing fish embryos have not detected desmosomes at this stage (present work and [16,17]). However, we showed that maternal transcripts for both zfDsc and zfDsgα were present from the start of development so these are clearly functional at this very early stage. (These early effects were obtained only with ATG morpholinos; maternal mRNA is spliced and as anticipated the splice site morpholinos were without effect (not shown).) The effects of knock-down were often quite severe, apparently involving detachment of superficial cells from the eYSL, which, in some cases continued epiboly movements. It has been shown previously in *Fundulus *that YSL movements continue independently after mechanical removal of the DC and EL [31]. Our results suggest the possibility that desmosomal cadherins may be involved in adhesion of the superficial layers to the eYSL.

The role of desmosomal cadherins in epiboly appears to differ from those described for two other adhesion molecules, E-cadherin and Ep-CAM, which have been shown to be functionally important during this stage, because the phenotypes generated by mutations and or knock-down of these molecules are different from those described here [1,2,5,17,32]. E-cadherin appears to be important for intercalation of interior layer cells into the exterior layer of the epiblast and/or adhesion of deep cells to the underside of the enveloping layer [1,2]. Ep-CAM is also involved in adhesion between deep cells and the enveloping layer as well as cell-cell adhesion within the enveloping layer [17]. No effects on epiboly were reported for knock-down of the desmosomal plaque armadillo-family protein plakoglobin, which occurs in adherens junctions as well as in desmosomes [33]. On the other hand morpholino knockdown of claudin-E, which is necessary for tight junction formation at the leading edge of the EVL, also caused a strong epiboly delay or arrest, similar to the phenotypes we have described here [34]. Interestingly, the claudin-E morpholino also caused a delay in eYSL epiboly.

Our ultrastructural studies show that while desmosomes were absent during early epiboly, they were present between cells of the enveloping layer at the shield stage, where they form the basal components of typical epithelial junctional complexes. This timing appears to correspond well with the appearance on zygotic desmosomal cadherin transcripts and is slightly earlier than "mid gastrulation" as reported for *Fundulus *by Trinkaus and Lentz [16]. Thereafter, desmosomes persisted between cells of the enveloping layer and by 24 hpf formed extensive arrays of ten or more individual junctions. Desmosomal cadherin knock-down was associated with the presence of fewer desmosomes and altered desmosome morphology, suggesting that desmosomal adhesion was reduced. (Ideally it would be desirable to confirm these observations by immunofluorescence and western blotting with appropriate antibodies. Screening of existing antibodies revealed none that reacted with zebrafish desmosomal cadherins and our attempts to raise such antibodies have so far proved unsuccessful.)

The changes in morphology that we have found in longer-surviving embryos namely, shortening of the embryonic axis, broadening of the pattern of *gata1 *expression and lateral enlargement of somites and of the field of *myoD *expression, are very similar to those found in a number of mutants that affect gastrulation and convergence-extension movements, including the mutant *half baked *which affect E-cadherin [2,35]. This suggests that the desmosomal cadherins also play an important role in coordinating these movements. Since electron microscopy suggests that desmosomes are confined to the junction complexes of the EL, it appears that maintenance of normal adhesion in these complexes is crucial for gastrulation and convergence-extension movements.

## Conclusion

Zebrafish have a single desmocollin, the orthologue of mammalian Dsc1, and two closely related desmogleins, orthologues of mammalian Dsg2. These are expressed early in zebrafish development where morpholino knockdown suggests that they play important role in epiboly, gastrulation and convergence-extension movements. This work provides a novel insight into zebrafish early development and a basis for further investigation of the role of desmosomal adhesion

## Competing interests

The authors declare that they have no competing interests.

## Authors' contributions

AG carried out embryo injection, phenotypic analysis, *in situ *hybridization, SYTOX injection studies and helped draft the manuscript. X-ML carried out cloning and sequencing, bioinformatics, embryo injection and phenotypic analysis. AH and DG conceived the study, participated in its design and coordination and drafted the manuscript.

## Supplementary Material

Additional file 1 **Sequences of zebrafish desmosomal cadherins**. The file contains the sequences of zfDsc, zfDsgα and zfDsgβ, and an amino acid identity comparison between zfDsgα and β. Click here for file
