# Correction: Desmosomal cadherins in zebrafish epiboly and gastrulation

**DOI:** 10.1186/1471-213X-14-13

**Published:** 2014-03-11

**Authors:** Alexander Goonesinghe, Xing-Ming Luan, Adam Hurlstone, David Garrod

**Affiliations:** 1 Faculty of Life Sciences, University of Manchester, Michael Smith Building, Oxford Road, Manchester M13 9PT, UK

## 

After the publication of this work [1], we were alerted to an error contained therein that we would hereby like to correct. In our study, we refer to two zebrafish paralogues of desmoglein (Dsg), which we label alpha and beta. We state in the text that they are encoded by ENSDARG00000062750 and ENSDARG00000076426 respectively. In truth, the protein sequence we describe as Dsgα is encoded by ENSDARG00000076945. ENSDARG00000062750 corresponding to position 16,990,224-17,010,459 on the reverse strand of chromosome 20 appears to encode a further Dsg paralogue, which we have not analysed any further.

We conclude that the zebrafish genome contains at least two and probably three genes encoding desmoglein paralogues.

We regret any inconvenience that this error might have caused. We wish to thank Dr. Alex Horby Christensen for bringing this matter to our attention.
