# Supplementary material for: Desmosomal cadherins in zebrafish epiboly and gastrulation
Source: BMC Dev Biol. 2012 Jan 11;12:1. doi: 10.1186/1471-213X-12-1 (PMC3268089; doi:10.1186/1471-213X-12-1)
Supplement: Additional file 1 — Sequences of zebrafish desmosomal cadherins. The file contains the sequences of zfDsc, zfDsgα and zfDsgβ, and an amino acid identity comparison between zfDsgα and β. [file 1471-213X-12-1-S1.DOCX]

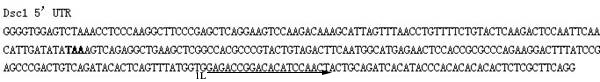


-284

-188

-92

1

1

97

33

193

65

297

97

375

129

471

161

567

193

663

225

759

257

855

289

951

321

1047

353

1143

385

1527

513

1623

545

1719

577

1239

417

1335

449

1431

481

1815

609

1911

641


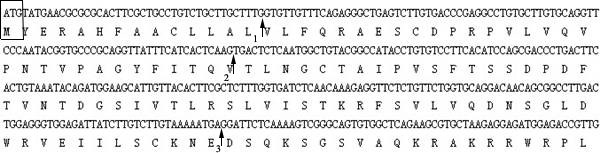

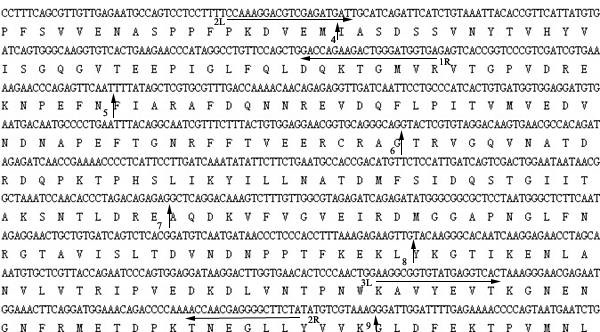

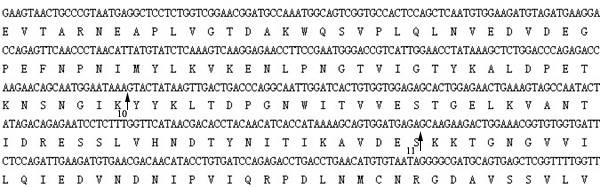

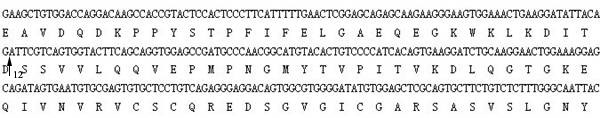


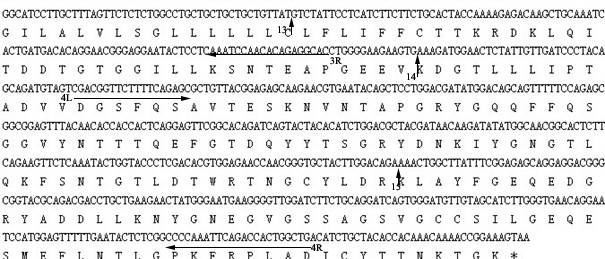

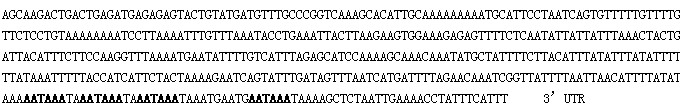


2687

2783

2879

2975

3071

2007

673

2103

705

2199

737

2295

769

2391

801

2487

833

2583

865

Figure S1 – zfDsc open reading frame and protein sequence

Schematic representation of full length zfDsc cDNA and protein sequence. The starting location of primer is marked by horizontal arrow. The end of each exon is marked by upright arrow. The box shows the start codon and the asterisk shows the stop codon. The TGA motif and polyadenylation signals are highlighted in the 5′ and 3′ UTR, respectively.


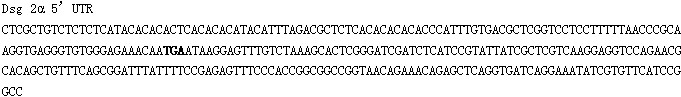


-287

-191

-95

-3

1

1

97

33

193

65

297

97

375

129

471

161

567

193

663

225

759

257

855

289

951

321

1047

353

1143

385

1239

417

1335

449

1431

481

1527

513

1623

545

1719

577

1815

609

1911

641

2007

673

2103

705

2199

737


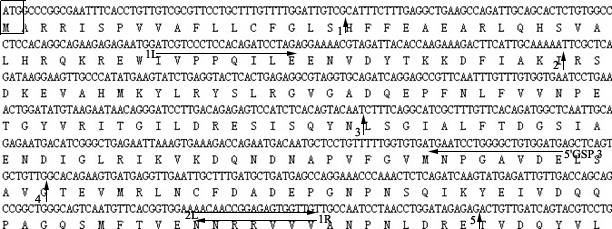


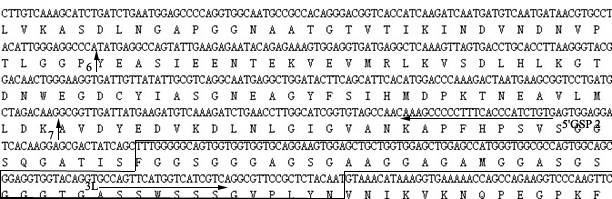


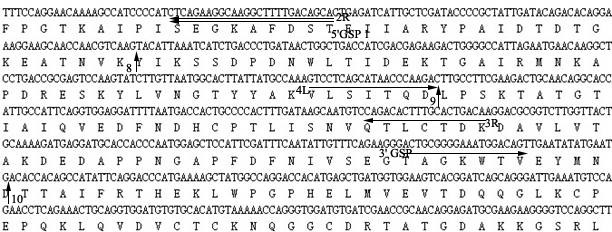


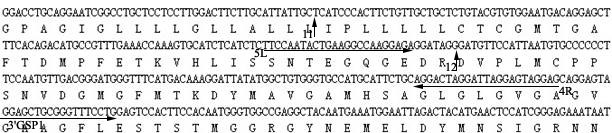


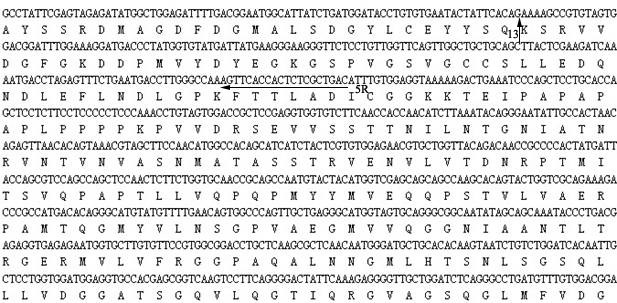


2295

769

2391

801

2487

833

2583

865

2679

897

2775

929

2871

961

2967

993

3063

1025

3159

1057

3255

1089

3351

1121

3420

3516

3612

3708


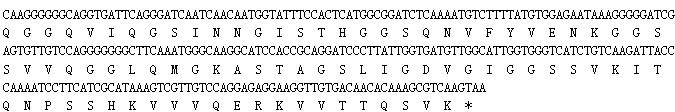


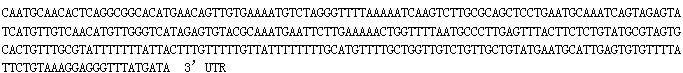


**Figure S2 – zfDsgα open reading frame and protein sequence**

Schematic representation of full length zfDsgα cDNA and protein sequence. The starting location of primer is marked by horizontal arrow. The end of each exon is marked by upright arrow. The box at the beginning shows the start codon and the asterisk showed the stop codon. The TGA motif was highlighted in the 5′. The frame in the middle shows a glycine rich segment and its cDNA sequence.

**
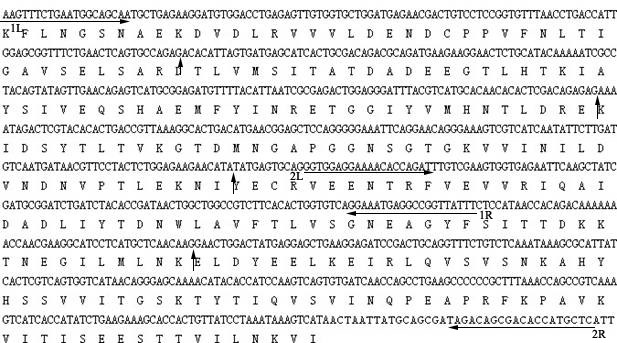
**

1

1

97

33

193

65

289

97

385

129

481

161

576

193

672

225

768

257

**Figure S3 – zfDsgβ partial open reading frame and protein sequence**

The exact locations of primers are marked by horizontal arrows. The upright arrows showed the boundaries of exons.

**Figure S4 – Amino acid identities of domains of zfDsgα and zfDsgβ from predicted protein sequences**
